# Psychological Correlates of Premenstrual Distress: The Role of Emotion Regulation and Menstrual Attitudes

**DOI:** 10.1192/j.eurpsy.2026.11041

**Published:** 2026-07-31

**Authors:** I. P. Rontogianni, A. Papadopoulou, S. Bargiota, P.-D. Stavrou, E. Vousoura, V. Efstathiou

**Affiliations:** 1 Department of Psychology, National and Kapodistrian University of Athens; 2 Second Department of Psychiatry, “Attikon” University General Hospital, National and Kapodistrian University of Athens, Athens; 3 Department of Psychiatry, University of Thessaly, Larissa, Greece

## Abstract

**Introduction:**

Premenstrual distress affects a substantial proportion of women, yet its psychological correlates remain underexplored. Emotion regulation and menstrual attitudes may play a key role in shaping premenstrual experiences.

**Objectives:**

This study aimed to examine the association of emotion regulation strategies and menstrual attitudes with self-reported premenstrual distress, premenstrual syndrome (PMS) and premenstrual dysphoric disorder (PMDD) classifications.

**Methods:**

A correlational design was employed with 572 cisgender women aged 18–46 (M = 27.5, SD = 7.3). Participants completed the Emotion Regulation Questionnaire, the Premenstrual Symptoms Screening Tool, and the Menstrual Attitude Questionnaire. Statistical analyses included correlation and mediation models.

**Results:**

Based on PSST screening, 51.7% of participants met criteria for PMS and 19.9% for PMDD. Premenstrual distress was positively associated with the Menstrual Attitude Questionnaire subscales *Menstruation as a Debilitating Event* and *Menstruation as a Bothersome Event*, and negatively associated with *Menstruation as a Natural Event* and *Anticipation and Prediction of Menstruation.* Within emotion regulation strategies, *Cognitive Reappraisal* was independently linked to lower distress, underscoring its protective role, whereas *Expressive Suppression* showed weaker associations. Mediation analysis further demonstrated that the subscale *Denial of Any Effect of Menstruation* partially explained the association between *Expressive Suppression* and distress: women who minimized or dismissed menstrual impact showed higher levels of distress when also relying on suppression. This suggests that *Denial of Any Effect of Menstruation* may serve as a mechanism through which *Expressive Suppression* contributes to premenstrual distress. No other menstrual attitude subscales significantly mediated this relationship.

**Conclusions:**

These findings underscore the critical role of emotion regulation and menstrual attitudes in shaping premenstrual experiences. They suggest that maladaptive attitudes, such as denial of menstrual impact, may exacerbate the negative effects of suppression, while cognitive reappraisal serves as a protective factor. Integrated mental health and medical approaches are needed to better support women with PMS and PMDD.

**Disclosure of Interest:**

None Declared

